# A North American, single-center experience implanting fenestrated atrial devices and atrial flow regulators into a heterogeneous group of pediatric pulmonary hypertension patients

**DOI:** 10.3389/fped.2023.1073336

**Published:** 2023-01-25

**Authors:** David Edward Youssef, Konstantin Averin, Susan Richards, Catherine Sheppard, Cameron Seaman, Matthew Pietrosanu, Angela Bates

**Affiliations:** ^1^ Division of Pediatrics, Department of Pediatric Pulmonary Hypertension, Stollery Children’s Hospital, Edmonton, AB, Canada; ^2^ Division of Pediatric Cardiology, Department of Pediatrics, University of Alberta, Edmonton, AB, Canada; ^3^ Cohen Children’s Heart Center, Donald and Barbara Zucker School of Medicine at Hofstra/Northwell, New Hyde Park, NY, United States; ^4^ Department of Mathematical and Statistical Sciences, University of Alberta, Edmonton, AB, Canada; ^5^ Division of Pediatric Critical Care, Department of Pediatrics, University of Alberta, Edmonton, AB, Canada

**Keywords:** atrial flow device, atrial septal defect, interventional procedures, hypertensive crisis, vasodilator therapy

## Abstract

**Introduction:**

The clinical deterioration commonly experienced by pediatric patients with pulmonary arterial hypertension (PAH) has motivated a shift in the treatment of pulmonary hypertension (PH) through innovations in surgical salvage interventions. The Occlutech fenestrated atrial septal defect (FASD) Occluder and the atrial flow regulator (AFR), which provides a protective, atrial-level shunt during hypertensive crises, have found an important role in treating pediatric patients with PAH. Other groups of pediatric patients with PH may also benefit from a similar protective physiology. The primary aim of this work is to present a single center's experience with AFR and FASD devices for managing a heterogeneous group of pediatric PH patients. A secondary goal is to identify hemodynamic changes and complications following device implantation.

**Materials and Methods:**

We performed a retrospective review of all pediatric PH patients who, after being found suitable, either successfully or unsuccessfully received an FASD or AFR device between January 2015 and December 2021 at the Stollery Children's Hospital in Edmonton, Canada.

**Results:**

Fourteen patients (eight female) with a median age of 4.6 (range 0.3–17.9) years and a median body mass index of 15.1 (Q_1_ = 13.8, Q_3_ = 16.8) kg/m^2^ underwent device implantation: five received FASDs, eight received AFRs, and one was ultimately unable to receive an implant due to thrombosed iliac vessels and required surgical intervention. Of the fourteen patients, seven were in group 1 (PAH), one was in group 3 (lung disease), and six were in group 5 (primarily pulmonary hypertension vascular disease) under the World Symposium PH classification. All patients were on mono-, dual-, or triple-drug PH therapy. Device stabilization was not possible for two patients, who then required a repeat catheterization. Of the group 1 patients, three AFR and three FASD implants were successful, while one FASD implant was unsuccessful due to thrombosed vessels. At a six-month clinical assessment, all group 1 patients had patent devices and improved WHO FCs.

**Conclusion:**

This work presents a single center's experience with AFR and FASD implants in a heterogeneous group of fourteen pediatric patients with severe PH. This treatment strategy is novel in the pediatric population and so this work provides momentum for future studies of interventional cardiac catheterization procedures for pediatric patients with PH. Further collaborations are required to develop criteria to identify ideal pediatric candidates and optimally time interventions in order to maximize the benefits of this treatment.

## Introduction

Pulmonary arterial hypertension (PAH) is a severe, irreversible, and progressive disease that involves destruction to the pulmonary vascular bed, increases in right-ventricular pressure, and eventual failure of the right ventricle. All these factors contribute to the disease's high long-term morbidity and mortality (1). Pivotal to the management of pulmonary hypertension (PH) are early and aggressive treatments that aim to improve patient quality of life and reduce mortality. Over the past few decades, the epidemiology of pediatric PH has been changing: while the rates of idiopathic and heritable PAH have remained stable, those for PAH associated with congenital heart disease, PAH secondary to developmental lung disease, and pulmonary hypertensive vascular disease (PHVD) have increased (2).

Despite advances in treatment options that target multiple pathways, many patients deteriorate even when on combination therapy. This leads to frequent and prolonged hospitalizations, a progressive decline in quality of life, and increased morbidity and mortality. Previous work in the Registry to Evaluate Early and Long-term PAH Disease Management (REVEAL) study estimated the five-year survival time from diagnosis for children under 18 years of age to be 74%; patients diagnosed at an older age had a worse prognosis (3). There has consequently been a shift toward aggressive medical management strategies (including the early use of dual and triple therapy) and greater need for treatment-resistant options for patients with PAH.

The advantages and disadvantages of an atrial septal defect (ASD) are often difficult to determine for pediatric PH patients. This determination can be even more challenging among group 3 patients with developmental lung disease, where even a small shunt might not be well tolerated by the vulnerable developing pulmonary vascular bed. For some patients, the benefit of closing an ASD to minimize excess left-to-right shunting is balanced by the protective effect of right-to-left shunting during pulmonary hypertensive crisis or syncope (4–6). Failure of the right ventricle is associated with acute elevation of pulmonary arterial pressure (PAP) from baseline. Increased PAP, especially if sudden and significant, further leads to an increase in right-ventricular end diastolic pressure (RVEDP) and a subsequent elevation of right-atrial pressure (RAP), dilation of the right ventricle, bowing of the interventricular septum into the left ventricle, and an increase in left-ventricular end diastolic pressure. Associated sequelae are signs and symptoms of acute right ventricle failure, decreased cardiac output, coronary artery perfusion, and cerebral perfusion leading to cardiac arrest (Supplementary Figure S1). The creation of a controlled atrial fenestration with an atrial flow regulator (AFR) can decrease RAP and offer a right-to-left shunt, increase cardiac output to the left side, and decrease RVEDP and right ventricle dilation. This strategy both offloads the right ventricle and improves systemic cardiac output with the consequence of mild desaturation (6).

Until recently, the only options available to children were surgical ASD closure with a residual fenestration; percutaneous closure with a modification of an existing device; or, for patients with an intact atrial septum, a balloon atrial septostomy. All of these approaches are suboptimal (3). The Occlutech atrial flow regulator (AFR) and the fenestrated ASD (FASD) Occluder allow for the creation or maintenance of stable interatrial communication in patients with PAH. These purpose-made devices allow for atrial communication or reduce the size of an ASD without the need for cardiopulmonary bypass or the downsides of modified percutaneous devices.

Reports on the implantation of these devices in the pediatric population are limited to the works noted in Supplementary Table S1. Even more scarce are reports on experiences with these devices among group 1 and group 5 patients, especially with regard to patient selection and timing. Growing experience suggests two types of vulnerable group 1 patients: those who benefit from an AFR, which creates an ASD and protects the right ventricle, and those who benefit from an FASD, which minimizes ASD size and allows pulmonary vasodilator therapy to be optimized. Nonetheless, the literature is still limited regarding patient selection and outcomes (7–12).

This work presents a single-center experience of a heterogeneous group of pediatric patients who underwent a catheter-based procedure with AFRs and FASDs. Herein we present patient demographics, PH diagnoses and etiologies, and acute- and mid-term outcomes following intervention. The primary aim of our work is to examine a single center's experience with AFR and FASD devices for managing pediatric PH. A secondary goal is to identify changes in hemodynamics and functional classification as well as complications following device implantation.

## Materials and methods

We present a retrospective, single-center experience of all PH patients who underwent ASD closure with an FASD or atrial shunt creation with an AFR between January 2015 and December 2021 at the Stollery Children's Hospital in Edmonton, Canada.

Demographic and clinical details including sex, age at device implantation, device type and size, clinical indications, PH classification, and genetic conditions were collected at baseline. Information on pulmonary vasodilator therapy regimens and additional medications at the time of intervention (including anticoagulants/antiplatelets), hemodynamic data prior to and immediately following device implantation, clinical characteristics, vital signs, results from the six-minute walk test, and echocardiographic parameters prior to implantation and at six months post-intervention were also collected.

### Decision pathway and patient eligibility

The decision pathway for the patients in this study made use of imaging data (e.g., echocardiography, computed tomography, cardiac magnetic resonance imaging), 12-lead electrocardiogram data, six-minute walk test results where appropriate, laboratory data (including that on NT-proBNP and liver function), and a medication review. All patients were discussed at a joint cardiac surgical conference with representation from cardiology, pediatric cardiothoracic surgery, and interventional cardiology. A patient was deemed to be suitable if the following criteria were met.

• A group 1 patient must have worsening PH symptoms including PH crises, syncope, an inability to augment pulmonary vasodilator therapy, or reduced exercise tolerance despite optimal PH vasodilator therapy. All patients were on optimal or maximal PH therapy.
• A group 5 patient (with single-ventricle physiology following cavopulmonary anastomosis or Fontan completion) must have remained desaturated and have evidence of elevated pulmonary vascular resistance or hypoxemia.

Patients were excluded if the joint decision was that the patient was unsuitable for implantation under the following criteria.

• Atrial communication was unfavorable based on multimodal imaging.
• The procedure was likely to be unsuccessful secondary to the probable etiology of PH.
• The risk of the procedure outweighed the benefit as determined by the multidisciplinary team.
• A patient was unsuitable based on atrial septal morphology or if a preoperative assessment medication review took place in association with a pediatric pharmacist and medication was optimized or modified.

### Technical details and follow-up

No patients received a cardiac catheterization prior to the joint cardiac surgical decision as multimodal imaging was employed for a cardiac assessment. Following a decision to proceed with implantation, a cardiac catheterization was performed under general anesthesia by a cardiac anesthetist familiar with managing pediatric PH patients. All patients received a preprocedure PH assessment by our PH team, cardiac anesthetists, and the pediatric cardiac intensive care unit. All patients, caregivers and parents were counseled by the members of the multidisciplinary team. Ample opportunity was allowed to assess, seek, and review any concerns related to the procedure.

A standard technique with a Brockenbrough trans-septal needle was utilized to perform a trans-septal puncture (typically a 7F Mullins trans-septal sheath) with subsequent static and cutting balloon angioplasty of the atrial septum to 150%–200% of the intended AFR size (e.g., pre-dilation to 8 mm for a 4 mm AFR implant). Final sheath size was determined by that required for the AFR device. Therapeutic heparin was used as a bridge to dual antiplatelet therapy for all devices.

Following device implantation, all patients received regular structured follow-ups that included clinical, imaging (echocardiographic), and laboratory assessments. Cardiac catheterization and hemodynamics were obtained under resting conditions with normal gas exchange (pH 7.35–7.45 and paCO_2_ 35–45 mmHg). Where possible, the following measurements were obtained:

• systolic, mean, and diastolic systemic arterial pressures;
• right-arterial pressure;
• right-ventricular systolic end-diastolic pressure;
• systolic, mean, and diastolic mean pulmonary arterial pressure (mPAP);
• pulmonary arterial wedge pressure (PAWP);
• left-atrial and left-ventricular end-diastolic pressure;
• transpulmonary gradients, simultaneous PAWP, and end-diastolic pressure; and
• cardiac index and pulmonary vascular resistance index *via* standardized formulas based on hemodynamic measurements (2, 13).

All patients, even those for whom devices were not successfully deployed, were included. As the devices used are not approved for commercial use by Health Canada, approval was granted through the Health Canada Special Access Program *via* an application for each patient. Ethics approval was obtained from the Health Research Ethics Board at the University of Alberta (Pro00116783).

### Statistical analysis

Statistical analyses were performed using R version 3.6.3 (14). Categorical data are summarized with counts and relative percentages, and numerical data with the first through third quartiles. Paired Wilcoxon signed-rank tests are used to compare pre- and post-implantation measures. Raw *p*-values are presented throughout. A significance level of 0.05 is used in all subsequent interpretations.

## Results

### Cohort characteristics

In total, fourteen patients (eight females and six males) received either an FASD or ASD between January 2015 and December 2021. Two patients underwent repeat catheterizations at different dates; these patients are each represented as a single record. See Supplementary Table S2 for more detail. Patients' clinical characteristics and hemodynamics at baseline are summarized in Table 1: median age at implantation was 4.6 (range 0.3–17.9) years, median body mass index was 15.1 (Q_1_ = 13.8, Q_3_ = 16.8) kg/m^2^_,_ median oxygen saturation at rest was 92.0% (Q_1_ = 85.5%, Q_3_ = 96.5%), and median baseline NT-proBNP was 2884.0 (Q_1_ = 1217.0, Q_3_ = 11,256.0) ng/l. For morphometric measures, diagnoses, clinical indications, therapies, and medications at the individual level, see Supplementary Table S2.

**Table 1:** Baseline demographics and clinical measures for All fourteen patients, presented as median (Q_1_, Q_3_) or count (%).

Variable/level	Summary
**Sex**	
Male	6 (42.9%)
Female	8 (57.1%)
**Age and anthropometrics**	
Age (years)	4.6 (1.9, 8.6)
Weight (kg)	16.4 (8.3, 22.2)
Height (cm)	107.8 (82.5, 124.3)
Body mass index (kg/m^2^)	15.1 (13.8, 16.8)
**WSPH classification**	
Group 1 (PAH)	7 (50.0%)
Idiopathic PAH	4 (57.1% of group 1)
PAH-CHD	3 (42.9% of group 1)
Group 3 (lung disease)	1 (7.1%)
Group 5 (PHVD)	6 (42.9%)
**Clinical/biomedical profile**	
O_2_ saturation (at rest) (%)	92.0 (85.5, 96.5)
NT-proBNP (ng/l)	2884.0 (1217.0, 11,256.0)

NT-proBNP, N-terminal prohormone B-type natriuretic peptide; PAH-CHD, pediatric arterial hypertension associated with congenital heart disease.

The cohort contained seven patients in group 1 (PAH) (four with idiopathic PAH and three with PAH associated with congenital heart disease), one in group 3 (lung disease), and six in group 5. The patients in group 5 predominantly had single-ventricle physiology with Fontan circulation and PHVD (transpulmonary gradient greater than 7 mmHg and/or pulmonary vascular resistance index above 3 iWU). The majority of these patients had hypoplastic left heart syndrome. One of the group 5 patients had pulmonary atresia with an intact ventricular septum and major aortopulmonary collateral arteries; an FASD device was inserted in a hypoxemic setting to minimize flow across the atrial septum. This patient was unique among the patients with single-ventricle indications. While this patient had PHVD by definition, the FASD was placed due to hypoxemia in the atrial septum position. One patient had trisomy 21 and one had DiGeorge syndrome (*22q11.21*–*q11.23*). There was one variant of unknown significance and one copy number variation (*12 p11.21*). All patients, except one who underwent successful device implantation, received anticoagulant therapy immediately after device deployment for shunt preservation. Five of the patients were on diuretic therapy both before and after device implantation.

### Difficult device implantations

Two patients each required two separate cardiac catheterizations. The device originally received by Patient 2 embolized shortly after deployment but was successfully retrieved and removed with no ill effects. Six months later, the patient returned and received a larger FASD implant. Patient 4's fenestration spontaneously closed shortly after the intervention and a larger device was implanted successfully at a later date.

Device deployment was unsuccessful for patients 13 and 14. Patient 13's device could not be implanted due to bilateral femoral venous occlusion. Device deployment for Patient 14 (five months old at the time of intervention) was not successful due to a moderate ASD: the device embolized to the left atrium and left ventricle on the first and second attempts, respectively. The device was successfully retrieved after each attempt and, ultimately, the infant received a surgical ASD closure.

### Clinical, hemodynamic, and echocardiographic impact

**Figure 1:**
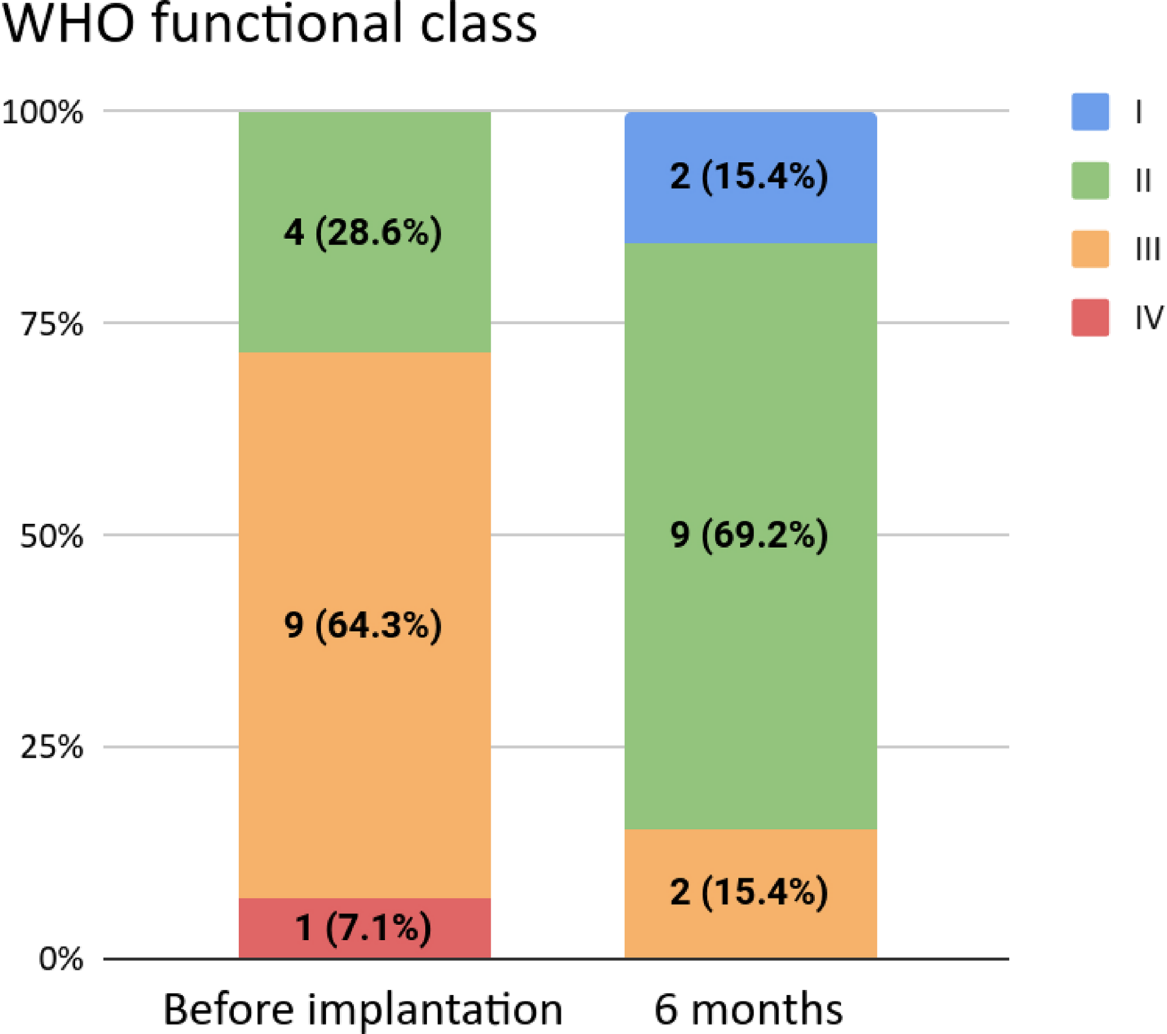
WHO functional classifications before and six months after implantation. The latter was not available for one patient.

We observed a statistically significant improvement in WHO functional classification (WHO FC) among all patients (*p* = 0.018): in our cohort, five patients (all class III pre-implantation) were reclassified into class II and two patients (one in each of classes III and IV pre-implantation) were reclassified into class I. The other patients remained in one of class II or III over the study period. No patients worsened with respect to WHO FC. The distributions of pre- and post-implantation WHO FC are shown in Figure 1 for all patients and in Table 2 for group 1 patients.

**Table 2:** Pre and post measures for the seven group 1 patients, presented as median (Q_1_, Q_3_) or count (%).

Variable	Pre measure^1^	Post measure^1^	*P*-value^2^
**Clinical/biochemical profile** ^3^			
NT-proBNP (ng/l)	2284.0 (2023.0, 5656.0)	-	-
SpO_2_ (at rest) (%)	97.0 (94.0, 99.5)	-	-
SpO_2_ (during exercise) (%)	93.0 (92.5, 93.5)	-	-
**Hemodynamics** ^4^			
CI (mL/min/m^2^)	3.8 (3.2, 4.2)	3.7 (3.0, 3.9)	0.25
mPAP (mmHg)	24.0 (24.0, 39.0)	30.5 (22.8, 37.3)	0.18
PCWP (mmHg)	7.0 (6.0, 8.0)	7.0 (5.8, 8.3)	1.00
PVRI (WU m^2^)	4.7 (4.6, 7.0)	5.1 (3.5, 7.4)	0.13
RAP (mmHg)	5.0 (4.0, 5.0)	3.0 (2.5, 3.5)	-
RVSp (mmHg)	40.0 (35.0, 54.0)	26.5 (25.3, 27.8)	-
**Echocardiography** ^5^			
PAAT (ms)	0.1 (0.1, 0.1)	0.1 (0.1, 0.1)	-
RVFAC (%)	38.3 (37.4, 39.8)	43.5 (40.4, 46.2)	0.59
RVSP (mmHg)	45.0 (39.0, 63.0)	54.0 (45.5, 54.5)	-
TAPSE (cm)	2.0 (1.8, 2.7)	1.9 (1.7, 2.2)	0.85
TV e′ (cm/s)	11.2 (9.1, 15.3)	13.0 (12.4, 15.2)	-
TV s’ (cm/s)	12.8 (11.4, 14.5)	11.1 (10.5, 11.1)	-
**WHO FC**			
Class I	0 (0.0%)	2 (33.3%)	0.18
Class II	2 (28.6%)	3 (50.0%)	
Class III	4 (57.1%)	1 (16.7%)	
Class IV	1 (14.3%)	0 (0.0%)	

CI, Cardiac index; NT-proBNP, N-terminal pro B-natriuretic peptide; PAAT, pulmonary arterial acceleration time; PCWP, pulmonary capillary wedge pressure; PVRI, pulmonary vascular resistance index; RAP, right-atrial pressure; RVFAC, right-ventricular fractional area change; RVSP, right-ventricular systolic pressure; TAPSE, tricuspid annular plane systolic excursion; TV e′ and TV s′ are indices of tissue doppler velocities of the tricuspid valve.

^1^ Clinical/biomedical measurements, echocardiographic measurements, and WHO FC were obtained at baseline and six months after implantation. Hemodynamic measurements were made before and immediately after implantation.

^2^ Comparisons are not conducted with two or fewer pre–post measurement pairs. For the available comparisons, *n* = 4 except for CI (*n* = 3) and WHO FC (*n* = 6).

^3^ For SpO_2_ (during exercise), *n* = 2. For SpO2 (at rest) and NT-proBNP, *n* = 7. No corresponding post data was available.

^4^ For pre hemodynamic measurements, *n* = 5 except for CI (*n* = 3). For post hemodynamic measurements, *n* = 2 for CI and RAP; *n* = 3 for CI; and *n* = 4 for mPAP, PCWP, and PVRI.

^5^ For pre echocardiogram measurements, *n* = 1 for PAAT, *n* = 6 for RVFAC, *n* = 5 for RVSP, *n* = 7 for TAPSE, and *n* = 4 for TV e′ and TV s′. For post echocardiogram measurements, *n* = 2 for PAAT, *n* = 4 for RVFAC, *n* = 3 for RVSP, *n* = 4 for TAPSE, and *n* = 3 for TV e′ and TV s′.

Significant pre–post differences in hemodynamic measures were not detected among group 1 PAH patients: these results are summarized in Table 2. A decrease in median right-ventricular systolic pressure (RVSP) from 40.0 mmHg to 26.5 mmHg was observed. Median cardiac index was comparable pre- and post-implantation at 3.8 L/m^2^ and 3.7 L/m^2^, respectively.

No statistically significant changes were detected in echocardiographic or tissue doppler imaging parameters between pre- and post-implantation for patients in group 1. We observed an increase in median RVSP from 45.0 mmHg to 54.0 mmHg and a decrease in median tricuspid annular plane systolic excursion (TAPSE) from 2.0 cm to 1.9 cm. We observed an improvement in median right-ventricular fractional area change (RVFAC) from 38.3% to 43.5%. These results are summarized in Table 2.

## Discussion

This work represents a single center's experience with AFRs and FASDs in a heterogeneous group of fourteen pediatric patients with severe PH, among which twelve had successful implantations. The two unsuccessful implantations were due to device embolization and thrombosed iliac vessels that prohibited femoral vascular access. The majority of the cohort had devices that were patent at six months post-implantation. We observed improvements in various echocardiographic measures, but none of these pre–post comparisons were statistically significant: we attribute this in part to the small size of the cohort and the lack of consistently available data. On the other hand, we observed a statistically significant improvement in WHO FC across the entire cohort: seven patients saw an improvement in their classification post-implantation. Thirteen of the patients were alive after a mean follow-up time of 17.4 months. This work is the largest single-center experience in children of AFRs and FASDs in the pediatric population: see Supplementary Table S1 for a summary of related literature.

Three factors are recognized as contributing to PH associated with ASDs: the size of the ASD, the location of the ASD, and associated syndromes and lesions. Interplay between the size of the defect and the compliance of the right ventricle predicts the potential for right-ventricular volume overload stemming from a left-to-right shunt (2). By Poiseuille's law, pulmonary pressure is the product of pulmonary blood flow and pulmonary vascular resistance (15). Therefore, in children with systemic-to-pulmonary shunts (i.e., ASDs), increased left-to-right shunting at the atrial level causes an increase in pulmonary blood flow, which subsequently leads to progressive vascular changes, increased shear stress, smooth muscular hypertrophy, and endothelial dysfunction (16). Although this is not the only contributing factor in the development of PAH, interventions such as AFRs and FASDs modify the elevation of PAP stemming from flow and shear stress and reduce the potential for further vascular remodeling. FASDs allow vasodilator therapy to modify disease progression and improve patient quality of life. In our cohort, the clinical benefits of AFR and FASD implantation were clinically (but not statistically) significant among patients in group 1. At follow-up, three of these patients were reported to be effectively utilizing their atrial shunts and reported fewer syncopal events relative to before implantation.

Group 5 (PHVD) patients require separate mention although they are not the primary focus of the current work. It is recognized that patients with Fontan physiology who have elevated cavopulmonary pressures have poorer survival outcomes and that the creation of a fenestration to augment elevated right ventricle or cavopulmonary pressures improves survival. However, this comes at the expense of lower systemic oxygen saturations. In addition, patients who have too large a fenestration and require a reduction in fenestration size to improve systemic oxygen saturations without a complete obliteration of the fenestration would also benefit from such a device. In our cohort, six patients had Fontan circulation. The role of the devices among the patients in group 5 included fenestration reduction in two cases, fenestration occlusion in two cases, and fenestration creation in two cases. While the focus of our work is not on this subgroup in particular [e.g., similar to the work by O’Callaghan et al. (17)], we noted improvements in oxygen saturation and WHO FC at follow-up. While in the work by O'Callaghan, five of the six pediatric patients had failing Fontans, our cohort only included one patient with a failing Fontan. Three of the six group 5 patients required closure or fenestration size reduction for systemic desaturation. Our work adds to the current literature regarding the unique and complex subset of group 5 patients with single-ventricle physiology. For these patients, the combination of pulmonary vasodilator therapy and interventional procedures as described above should in theory improve survival.

In adherence to current guidelines from the American Heart Association and American Thoracic Society, the patients in our cohort were provided with individualized treatments. Each patient was considered from a multidisciplinary perspective and received recommendations from a cardiologist, a team of PH specialists, and an interventional cardiologist. An individualized approach was required for each patient prior to choosing the device to implant.

Current literature on the use of AFRs and FASDs is limited to small case series and case reports, as shown in Supplementary Table S1. Kaley et al. (18) considers thirty-five device implantations, including six for pediatric patients with severe PAH. Of those patients, four were on dual therapy and two were on triple therapy. There was a 93% deployment success rate in their cohort. One pediatric mortality was reported secondary to progressive PAH one month after successful device implantation. At immediate and long-term follow-up, the other five children were observed to have symptoms consistent with the New York Heart Association's (NYHA's) class II categorization without the recurrence of syncopal episodes as well as an improvement in six-minute walk test results. Rajeshkumar et al. (19) considers twelve patients (aged 15–39 years) and demonstrates the benefits of atrial septostomy using AFR devices for patients on dual therapy (phosphodiesterase type-5 inhibitors and endothelin antagonists). The authors noted significant improvements in symptoms, six-minute walk test distance, cardiac index, and systemic oxygen transport. This is consistent with our study, which found patent atrial shunts and improvements in WHO FC. While we observed improvements in echocardiographic measurements post-implantation, we could not establish the statistical significance of these differences: we attribute this to the size of and availability of data within our cohort.

This is one of the first studies to describe an experience with AFRs and FASDs in a group of pediatric patients with severe PH. Although we observed improvements in objective and subjective parameters, the long-term benefits of this therapy are yet to be conclusively established. We recognize the limitations of this retrospective study, which include the small sample size, the lack of a comparator arm, and the heterogeneous patient group. As such, further work and collaboration (e.g., among international registries) is needed to increase knowledge and understanding of the role of atrial devices in managing pediatric PAH. Future work such as prospective, multicenter collaborations should consider the problem of identifying pediatric patients who should undergo device implantation and the optimal timing of these interventions. While the current work is retrospective, the decisions regarding individual patients are not known to the authors of the present work. While it would be insightful to know the decision algorithm for each individual patient, this information was not available for this retrospective review. Further prospective work is needed to guide future work.

Due to the technical details and the high level of expertise needed, the interventions described in this work should ideally be performed in pediatric PH centers given the high risk and potential for PH patients to decompensate with minimal provoking stimulus. Further details regarding technical parameters, including access sites, the complexities of implantation during deployment, and procedures to cross the atrial septum, go beyond the scope of the current work. Further multicenter and collaborative work is planned to explore the utility and potential of this procedure in the pediatric population.

## Conclusion

This work presents a single center's experience with AFRs and FASDs in a heterogeneous group of fourteen pediatric patients with severe PAH and demonstrates the range of PAH patients that can benefit from these devices. Among group 1 patients, clinical improvements in objective and subjective parameters were evident at six months following the intervention. Further multicenter work is required to develop criteria for identifying ideal pediatric candidates and set optimal intervention timing in order to maximize the clinical and symptomatic benefits of this treatment.

## Data Availability

The original contributions presented in the study are included in the article/**Supplementary Material**, further inquiries can be directed to the corresponding author/s.
