# Supplementary material for: A North American, single-center experience implanting fenestrated atrial devices and atrial flow regulators into a heterogeneous group of pediatric pulmonary hypertension patients
Source: Front Pediatr. 2023 Jan 25;11:1073336. doi: 10.3389/fped.2023.1073336 (PMC9905673; doi:10.3389/fped.2023.1073336)
Supplement: Supplementary file 1 [file Datasheet1.pdf]

Supplementary Material

1 **Supplementary Figure 1.** Pathophysiology of the creation of a rescue right-to-left shunt.

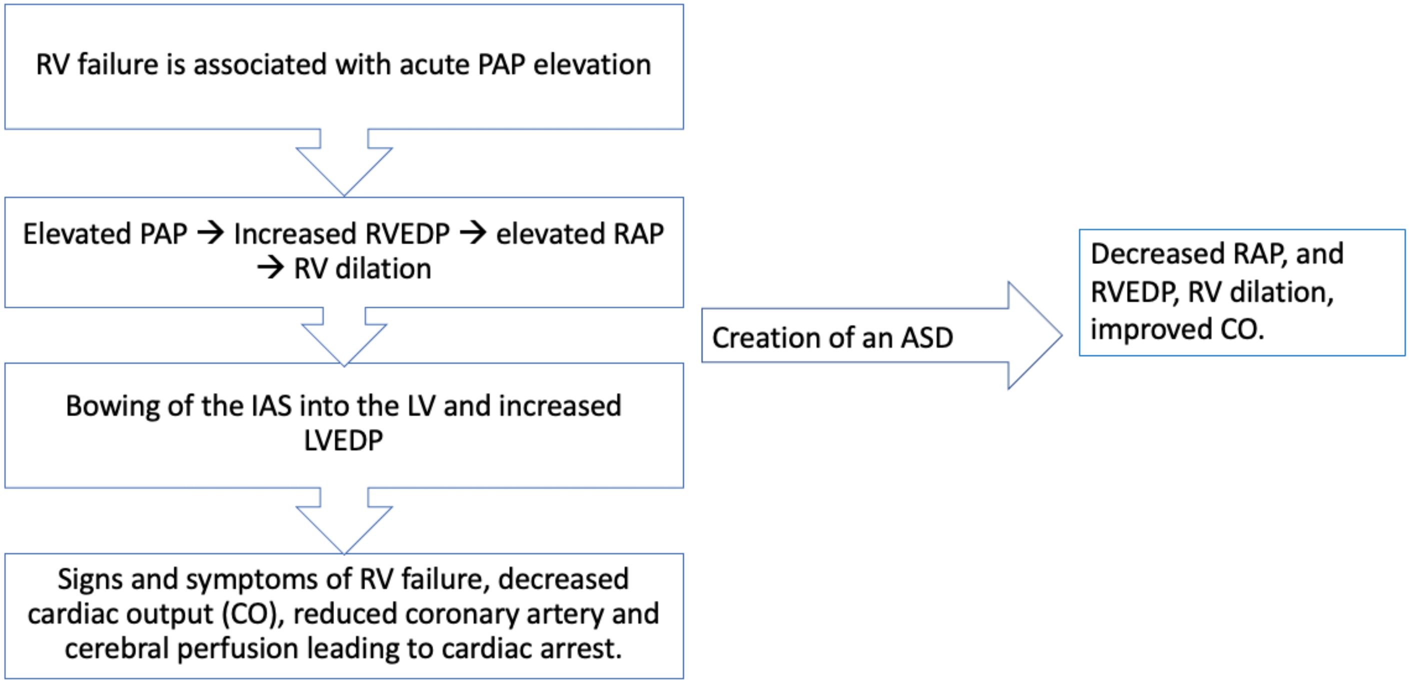

Note. ASD, atrial septal defect; CO, cardiac output; IAS, interatrial septum; LV, left ventricle; LVEDP, left-ventricular end diastolic pressure; PAP, pulmonary arterial pressure; RAP, right atrial pressure; RV, right ventricle; RVEDP, right-ventricular end diastolic pressure

Supplementary Table 1. Summary of Data Available in the Literature on Atrial Flow Regulator Implantation for Pediatric Pulmonary Arterial Hypertension Patients

Reference	Study details	n	Symptoms	Complications	Follow-up	Outcomes
Patel et al. (2015) (1)	Implantation of an AFR for a patient with PAH	1	- NYHA class III<br>- Impaired 6MWT<br>- Progressive ascites and pedal edema	None	6 weeks	- Improvement in 6MWT<br>- Relief of ascites and pedal edema<br>- Subjective symptomatic improvement<br>- Mean resting saturation 98%
Rajeshkumar et al. (2017) (2)	AFR devices implanted in patients with severe PAH presenting with syncope and right heart failure	12	- NYHA class III (9/12)<br>- NYHA class IV (3/12)<br>- Syncope or presyncope (12/12)<br>- Angina (3/12)<br>- Palpitations (3/12)	- Preprocedural atrial flutter (1/12)<br>- Postprocedural hypoxia warranted continued oxygen supplementation for 48–72 hours (6/12)	Median 189 (range 10–296) days	- Improvement in NYHA class (12/12)<br>- Relief of syncope (12/12)<br>- Subjective symptomatic improvement in 6MWT<br>- Preserved device patency (12/12)<br>- Mean resting saturation 92%
Dąbrowska-Kugacka et al. (2019) (3)	AFR for severe, drug-resistant PAH after CHD correction	1	- WHO class IVa<br>- Recurrent syncope<br>- Ascites and pedal oedema	None	6 weeks	- Improved WHO class<br>- Relief of syncope<br>- Relief of edema

Janus et al. (2020) (4)	AFR as a bridge to lung transplant in a young patient with drug-resistant, idiopathic PAH	1	- WHO class IV<br>- Impaired 6MWT<br>- Ascites and pedal edema	Postprocedural hypoxia warranting O_2	8 weeks	- Improved WHO class<br>- Improved 6MWT
Hansmann et al. (2022) (5)	Implantation of AFR devices in 3 children (age 6–13 years)	3	- WHO FCs of III–IV<br>- 1 patient had dyspnea on exertion, edema, and ascites<br>- 1 patient was listed for heart transplantation for 2 years prior<br>- All patients had group 2 PH secondary to RCM	Unknown: not reported, short follow-up time	1–3 months	Improved left-atrial dilation, postcapillary pulmonary hypertension, and heart failure symptoms
O'Callaghan et al. (2022) (6)	Implantation of AFR devices in 15 patients with greatly varying age	6 pediatric, 9 adult	- Failing Fontan (5/6)<br>- 1 patient had Shone's complex and extensive surgical history; unable to wean from ventilation and had left atrial hypertension	- 3/15 died (1 after major hemorrhagic stroke on ECMO)<br>- 2/15 patients with CHD and PH died from complications related to right heart failure	5 weeks (3-8)	- O_2 saturations increased in all Fontan patients<br>- Improvement in NYHA classification<br>- All patients survived to follow up

Note. 6MWT, six-minute walk test; AFR, atrial flow regulator; CHD, congenital heart disease; ECMO, extracorporeal membrane oxygenation; NYHA, New York Heart Association; PAH, pulmonary arterial hypertension; PH, pulmonary hypertension; RCM, restrictive cardiomyopathy

Supplementary Table 2. Patient-level Demographic and Clinical Characteristics at Baseline

No.	Device: size	Sex	Age (years)	Weight (kg)	Primary diagnosis	Clinical indication for devices	PH classification	Genetics	PH therapy	Anticoagulants/ other medications
1	FASD: 21 mm (5 mm fenestration)	F	9.35	70.0	APAH-CHD (large secundum ASD)	Orthopnea, effort intolerance	1.4	Nil	Tadalafil	Aspirin, Coumadin
2[1]	FASD: 24 mm (6 mm fenestration)	M	10.03	28.0	IPAH (incidental large ASD)	Dyspnea, fatigue, unable to augment vasodilator therapy	1.1	Nil	Ambrisentan, selexipag, tadalafil	Aldactazide, clopidogrel
3	AFR: 4 mm Occlutech	M	3.52	15.0	Fontan (DORV/MA)	Reduction in fenestration	5.4	Nil	Sildenafil	Amlodipine, Coumadin, enalapril
4[2]	AFR: 6 mm	F	4.38	13.9	Fontan (HLHS: AS/MA)	Fontan failure	5.4	Nil	Sildenafil	Aspirin, enalapril, enoxaparin, furosemide, spironolactone
5	AFR: 4 mm	M	14.59	19.9	Fontan (HLHS: AS/MA)	Occlusion of Fontan fenestration	5.4	Nil	Tadalafil	Coumadin
6	AFR: 6 mm	F	4.76	20.8	Fontan (HLHS: AS/MA)	Occlusion of Fontan fenestration	5.4	Nil	Ambrisentan, tadalafil	Coumadin
7	AFR: 6 mm	M	6.29	22.7	IPAH	Syncopal events, PH crises	1.1	Nil	Ambrisentan, tadalafil, remodulin	Aldactazide, aspirin, clopidogrel, Keppra
8	AFR: 4 mm	F	6.15	17.8	Fontan (HLHS: AS/MS)	Elevated PVRI	5.4	Nil	Ambrisentan, tadalafil	Coumadin
9	FASD: 10.5 mm	F	0.31	6.8	APAH-CHD (large PDA, moderate-to-large ASD)	CLD, persistent NIV requirement	1.4	T21	Tadalafil	Nil

10	AFR: 4 mm	F	1.86	7.9	APAH-CHD (hemitruncus: RPA from AA, large PDA to LPA)	PH crises requiring reliable atrial shunt	1.4	VOUS	Ambrisentan, tadalafil, treprostinil	Aspirin, clopidogrel, furosemide, spironolactone
11	FASD: 10.5 mm	M	1.92	9.5	Fontan (PA/IVS/MAPCA)	Multiple fenestrated ASD, hypoxemia	5.4	Nil	Tadalafil	Atenolol, enoxaparin
12	AFR: 6 mm	F	17.94	47.8	IPAH	Syncopal events, PH crises	1.1	DiGeorge syndrome	Ambrisentan, selexipag, tadalafil	Amlodipine, aspirin, enoxaparin
13[3]	[Failed due to iliac vessel occlusion; surgical closure]	F	0.40	5.3	APAH-CHD (large ASD)	Large shunt and prolonged intubation period	1.4	CNV *12p11.21*	Bosentan, tadalafil	Nil
14[3]	[Failed; 7.5 mm FASD embolized]	M	0.42	4.0	BPD-PH (moderate ASD, PDA)	Prolonged respiratory support, FTT	3.7	Nil	Ambrisentan, tadalafil	Lasix, spironolactone

Note. AA, ascending aorta; AA/MS, aortic atresia/mitral stenosis; AFR, atrial flow regulator; AP, aortopulmonary collateral; APAH-CHD, PAH associated with congenital heart disease; AS/MA, aortic stenosis/mitral atresia; AS/MS, aortic stenosis/mitral stenosis; ASD, atrial septal defect; BPD, bronchopulmonary dysplasia; CHD, congenital heart disease; CLD, chronic lung disease; CNV, copy number variation; DORV/MA, double outlet right ventricle/mitral atresia; F, female; FASD, fenestrated atrial septal defect; FTT, failure to thrive; HLHS, hypoplastic left heart syndrome; IPAH, idiopathic pulmonary arterial hypertension; IVS, interventricular septum; LPA, left pulmonary artery; M, male; MAPCA, major aortopulmonary collateral artery; NIV, noninvasive ventilation; PA/IVS, pulmonary atresia/intact ventricular septum; PAH, pulmonary arterial hypertension; PDA, patent ductus arteriosus; PH, pulmonary hypertension; PM, pacemaker; PS, pulmonary stenosis; PVRI, pulmonary vascular resistance index; RPA, right pulmonary artery; T21, trisomy 21; VOUS, variant of unknown significance

[1] Patient 2 has two cardiac catheterization events: the first device embolized and the second was deployed successfully six months later.

[2] Patient 4 had two cardiac catheterizations one month apart: the first attempt used a 4 mm AFR that was undersized for the atrial shunt while the second used a 6 mm AFR and was successful.

[3] Patients 13 and 14 did not receive anticoagulants after the procedure as devices were unable to be implanted.

References

1. Patel MB, Samuel BP, Girgis RE, Parlmer MA, Vettukattil JJ. Implantable atrial flow regulator for severe, irreversible pulmonary arterial hypertension. EuroIntervention (2015) 11(6):706–9.

2. Rajeshkumar R, Pavithran S, Sivakumar K, Vettukattil JJ. Atrial septostomy with a predefined diameter using a novel Occlutech atrial flow regulator improves symptoms and cardiac index in patients with severe pulmonary arterial hypertension. Catheterization and Cardiovascular Interventions (2015) 90(7):1145–53.

3. Dąbrowska-Kugacka A, Ciećwierz D, Żuk G, Fijałkowski M, Ottowicz A, Kwiatkowska J et al. Atrial flow regulator for severe drug resistant pulmonary arterial hypertension after congenital heart defect correction. Cardiology Journal (2019) 26(1):102–4.

4. Janus M, Sawek-Szmyt S, Araszkiewicz A, Mularek-Kubzdela T, Lesiak M, Grygier M. Atrial flow regulator as a bridge to lung transplant in a young patient with drug-resistant idiopathic pulmonary arterial hypertension. Kardiologia Polska (2020) 78(5):461–2.

5. Hansmann G, Sabiniewicz A, Sabiniewicz R. Atrial flow regulator for postcapillary pulmonary hypertension. JACC: Case Reports (2022) 4(14):878–84.

6. O'Callaghan B, Zablah J, Vettukattil J, Levi D, Salem M, Cabalka A et al. Multi-institutional US experience of the Occlutech AFR device in congenital and acquired heart disease. Congenital Heart Disease (2022) 17(1):107–16.
